# Supplementary material for: The Impact of Telemedicine on Parkinson’s Care during the COVID-19 Pandemic: An Italian Online Survey
Source: Healthcare (Basel). 2022 Jun 8;10(6):1065. doi: 10.3390/healthcare10061065 (PMC9222237; doi:10.3390/healthcare10061065)
Supplement: Supplementary file 1 [file healthcare-10-01065-s001.zip › Survey_Neurologist.pdf]

L'IMPATTO DEL COVID-19 SULL'USO DELLA TELEMEDICINA NELLA CURA DEI DISORDINI DEL MOVIMENTO

Gentile dottore,
siamo un team di psicologi del Policlinico di Milano e sociologi dell'Università Cattolica impegnati nella ricerca di nuovi modelli sanitari capaci di supportare adeguatamente i pazienti con disordini del movimento anche in questo momento di emergenza Covid-19.
Durante questo periodo, per ridurre gli accessi in ospedale e le possibilità di contagio, molti controlli neurologici in presenza sono stati convertiti in visite da remoto attraverso differenti modalità (Telefono, Email, SMS, Chat, Videoconsulto).
Ti chiediamo solo 10 minuti del tuo tempo per avere la tua opinione sulla qualità dei consulti neurologici a distanza erogati durante questo periodo pandemico. La tua partecipazione è volontaria, in qualsiasi momento potrai interrompere la compilazione senza dover fornire alcuna giustificazione.
I dati che ti riguardano saranno ritenuti come strettamente confidenziali e soggetti ad anonimato, i risultati potranno essere portati a conoscenza di terzi o pubblicati, ma escludendo ogni possibile riferimento personale. Ci si atterrà in ogni caso alle disposizioni normative vigenti relative alla tutela della privacy del soggetto (art.11 D.Lgs. 196/2003 – Codice in materia di protezione dei dati personali e all'art. 13 del GDPR 679/16 – "Regolamento europeo sulla protezione dei dati personali").

Proseguendo con la compilazione, acconsenti al trattamento dei dati
(se non acconsenti, non potrai partecipare alla ricerca)

- ○ Acconsento
- ○ Non acconsento

Età

____

Genere

- ○ Maschio
- ○ Femmina

Regione in cui lavori:

- ○ Abruzzo
- ○ Basilicata
- ○ Calabria
- ○ Campania
- ○ Emilia-Romagna
- ○ Friuli Venezia Giulia
- ○ Lazio
- ○ Liguria
- ○ Lombardia
- ○ Marche
- ○ Molise
- ○ Piemonte
- ○ Puglia
- ○ Sardegna
- ○ Sicilia
- ○ Toscana
- ○ Trentino-Alto Adige
- ○ Umbria
- ○ Valle d'Aosta
- ○ Veneto

Luogo di lavoro

- Università
- Ospedale Pubblico
- Ospedale Privato
- Studio Privato
- Altro (specificare) ____________________

Anni di esperienza lavorativa

- 0-5 anni
- 6-15 anni
- 16-25 anni
- 26-35 anni
- > 35 anni

Con quanti colleghi hai lavorato costantemente nell’ultimo anno?

- Da solo/a
- 1 - 3
- 4 - 10
- > 10

Hai prestato servizio in un reparto Covid-19?

- ◯ Sì
- ◯ No

Hai mai fornito un consulto neurologico a distanza prima della pandemia Covid-19 (per es. telefono, SMS, Chat, E-mail, Videoconsulto)?

- ◯ Sì
- ◯ No

Durante la pandemia Covid-19, hai avuto la possibilità di fornire soluzioni alternative alla visita neurologica in presenza (per es. telefono, SMS, Chat, E-mail, Videoconsulto)?

- ◯ Sì
- ◯ No

Chi ti ha informato della possibilità di erogare una visita neurologica a distanza?

- ◯ L’Ospedale per cui presto servizio
- ◯ È stata una mia proposta
- ◯ Me lo ha proposto il paziente

Hai utilizzato una piattaforma istituzionale per erogare il consulto neurologico a distanza?

- ◯ Sì
- ◯ No

Hai ricevuto assistenza tecnica/training per erogare il consulto neurologico a distanza?

- ◯ Sì
- ◯ No

Se si, in che misura è stato utile?

- ◯ Per nulla
- ◯ Leggermente
- ◯ Un po’
- ◯ Molto
- ◯ Moltissimo

Hai ricevuto delle linee guida istituzionali per erogare il consulto?

- ◯ Sì
- ◯ No

Se si, in che misura sono state utili?

- ○ Per nulla
- ○ Leggermente
- ○ Un po’
- ○ Molto
- ○ Moltissimo

Durante la pandemia Covid-19, per quali problematiche ti hanno richiesto assistenza più frequentemente? (possibili più risposte)

- ☐ Visita di controllo periodico
- ☐ Prescrizioni farmacologiche
- ☐ Regolazione dei parametri DBS
- ☐ Comparsa di effetti collaterali ai farmaci (dispercezioni visive, alterazioni del comportamento, etc.)
- ☐ Altro (specificare....) ____

Quali strumenti digitali hai utilizzato per erogare il consulto neurologico a distanza? (possibili più risposte)

- ☐ Telefono
- ☐ Email
- ☐ SMS
- ☐ Chat
- ☐ Videoconsulto es. videochiamata in WhatsApp, Skype, Zoom, Microsoft Teams
- ☐ Altro ____

ORA TI CHIEDIAMO DI FOCALIZZARTI SULLA TUA ESPERIENZA DI VIDEOCONSULTO E DI PENSARE AGLI ASPETTI TECNICI. ESPRIMI IL TUO GRADO DI ACCORDO/DISACCORDO CON LE SEGUENTI AFFERMAZIONI

	Per nulla d'accordo	Abbastanza in disaccordo	Né in accordo né in disaccordo	Abbastanza d’accordo	Molto d'accordo
1. Ritengo di avere le competenze informatiche necessarie	○	○	○	○	○
2. Ritengo di essere in possesso di strumentazioni digitali adeguate	○	○	○	○	○
3. Ritengo di aver ricevuto tutte le istruzioni necessarie per l’erogazione del consulto (password, link, altro)	○	○	○	○	○
4. Ritengo che sia stato semplice concordare un appuntamento con il paziente	○	○	○	○	○
5. La qualità dell’audio durante il consulto neurologico è stata adeguata	○	○	○	○	○

6. La qualità del video durante il consulto neurologico è stata adeguata	○	○	○	○	○
7. Ogni volta che ho riscontrato problemi tecnici ho risolto facilmente e rapidamente	○	○	○	○	○
8. È stato semplice usare i sistemi digitali per erogare il consulto	○	○	○	○	○
9. È stato facile imparare a usare i sistemi digitali per il consulto	○	○	○	○	○
10. Credo che potrei diventare sempre più esperto utilizzando servizi digitali per erogare prestazioni sanitarie	○	○	○	○	○
11. Ritengo che la privacy del paziente sia stata rispettata	○	○	○	○	○

ORA PENSA ALLA TUA ESPERIENZA DI VIDEOCONSULTO NEUROLOGICO A DISTANZA ED ESPRIMI IL GRADO DI ACCORDO/DISACCORDO CON LE SEGUENTI AFFERMAZIONI

	Per nulla d'accordo	Abbastanza in disaccordo	Né in accordo né in disaccordo	Abbastanza d'accordo	Molto d'accordo
12. Mi ha permesso di risparmiare tempo riuscendo a visitare un numero maggiore di pazienti	○	○	○	○	○
13. Mi ha permesso di ridurre i tempi di attesa per l’erogazione delle visite	○	○	○	○	○
14. È stato di grande utilità per i pazienti consolidati e per i follow-up di routine	○	○	○	○	○
15. Non sostituisce la visita in presenza ma può completarla	○	○	○	○	○
16. Rappresenta un'opzione importante e utile per l’assistenza clinica	○	○	○	○	○
17. Attraverso il consulto a distanza sono riuscito a spiegare adeguatamente le indicazioni farmacologiche che ho prescritto	○	○	○	○	○

18. Attraverso il consulto a distanza sono riuscito a spiegare adeguatamente la condizione clinica al paziente	○	○	○	○	○
19. Ritengo che questo sistema mi abbia permesso di fornire complessivamente informazioni sufficienti al paziente	○	○	○	○	○
20. Quando sarà finita l’emergenza sanitaria vorrei continuare ad usufruire di tale servizio	○	○	○	○	○
21. Lo consiglierei ad altri colleghi	○	○	○	○	○
22. Dovrebbe diventare parte integrante della pratica clinica ordinaria per l’assistenza sanitaria in futuro	○	○	○	○	○
23. Mi sono sentito/a a mio agio nel comunicare con il paziente a distanza	○	○	○	○	○
24. Ritengo che questo sistema mi abbia permesso di essere sufficientemente accurato nel corso della visita	○	○	○	○	○

PENSA ALL’INTERAZIONE CHE HAI AVUTO CON IL PAZIENTE DURANTE IL VIDEOCONSULTO ED ESPRIMI IL GRADO DI ACCORDO/DISACCORDO CON LE SEGUENTI AFFERMAZIONI

	Per nulla d'accordo	Abbastanza in disaccordo	Né in accordo né in disaccordo	Abbastanza d'accordo	Molto D'accordo
25. È stato facile esprimermi in modo efficace con il paziente	○	○	○	○	○
26. Ho potuto comprendere chiaramente quello che mi diceva il paziente	○	○	○	○	○
27. Sono riuscito a relazionarmi con il paziente come se fossimo in una visita in presenza	○	○	○	○	○
28. È stato confortevole erogare un consulto neurologico a distanza	○	○	○	○	○
29. Mi sono sentito/a a mio agio nell’utilizzare gli strumenti digitali	○	○	○	○	○
30. Mi sono sentito/a a mio agio nell’interagire con il paziente	○	○	○	○	○

31. Mi ha dato la possibilità di instaurare una buona relazione con il paziente	○	○	○	○	○
32. Mi ha permesso di dedicare un tempo adeguato all’ascolto dei problemi del paziente	○	○	○	○	○

PENSA ALLA TUA ESPERIENZA DI CONSULTO NEUROLOGICO A DISTANZA ED ESPRIMI IL TUO GRADO DI SODDISFAZIONE RISPETTO ALLE SEGUENTI DIMENSIONI

	Per nulla soddisfatto	Abbastanza insoddisfatto	Né soddisfatto né insodisfatto	Abbastanza soddisfatto	Molto soddisfatto
33. Qualità complessiva del servizio erogato	○	○	○	○	○
34. Attrezzatura digitale da me utilizzata	○	○	○	○	○
35. Attrezzatura utilizzata dal paziente	○	○	○	○	○
36. Puntualità del servizio erogato	○	○	○	○	○
37. Utilità nel fornire assistenza sanitaria	○	○	○	○	○
38. Tempo intercorso tra la prenotazione ed il consulto	○	○	○	○	○

TI CHIEDIAMO ORA DI PENSARE ALLE DIFFERENZE TRA VISITA NEUROLOGICA IN PRESENZA E CONSULTO NEUROLOGICO A DISTANZA. IN PARTICOLARE, CI INTERESSA CAPIRE PER OGNI ASPETTO PRESO AD ESAME SE SIA MEGLIO UN SERVIZIO IN PRESENZA, IL CONSULTO ONLINE OPPURE SE RITIENI NON CI SIANO DIFFERENZE

	Meglio visita in presenza	Meglio online	Non ci sono differenze
39. Accuratezza della visita	○	○	○
40. Prescrizioni farmacologiche	○	○	○
41. Durata della visita	○	○	○
42. Puntualità della visita	○	○	○
43. Possibilità di fare domande al paziente	○	○	○
44. Facilità di comprensione di quello che dice il paziente	○	○	○
45. Rispetto della privacy	○	○	○
