# Supplementary material for: The Impact of Telemedicine on Parkinson’s Care during the COVID-19 Pandemic: An Italian Online Survey
Source: Healthcare (Basel). 2022 Jun 8;10(6):1065. doi: 10.3390/healthcare10061065 (PMC9222237; doi:10.3390/healthcare10061065)
Supplement: Supplementary file 1 [file healthcare-10-01065-s001.zip › Survey_Patient.pdf]

Teleconsulto lato paziente

L’IMPATTO DEL COVID-19 SULL’USO DELLA TELEMEDICINA NELLA CURA DEI DISORDINI DEL MOVIMENTO

Caro partecipante,
siamo un team di psicologi del Policlinico di Milano e sociologi dell’Università Cattolica impegnati nella ricerca di nuovi modelli sanitari per le malattie croniche neurologiche, come la malattia di Parkinson e le sindromi parkinsoniane atipiche, capaci di supportare adeguatamente i pazienti anche in questo momento di emergenza Covid-19. Durante questo periodo, per ridurre gli accessi in ospedale e le probabilità di contagio, molti controlli neurologici in presenza sono stati convertiti in visite da remoto attraverso differenti modalità (Telefono, Email, SMS, Chat, Videoconsulto). Ti chiediamo solo 10 minuti del tuo tempo per avere la tua opinione sulla qualità dei consulti neurologici a distanza ricevuti durante questo periodo pandemico. La tua partecipazione è volontaria, in qualsiasi momento potrai interrompere la compilazione senza dover fornire alcuna giustificazione.
I dati che ti riguardano saranno ritenuti come strettamente confidenziali e soggetti ad anonimato, i risultati potranno essere portati a conoscenza di terzi o pubblicati, ma escludendo ogni possibile riferimento personale. Ci si atterrà in ogni caso alle disposizioni normative vigenti relative alla tutela della privacy del soggetto (art.11 D.Lgs. 196/2003 – Codice in materia di protezione dei dati personali e all'art. 13 del GDPR 679/16 – “Regolamento europeo sulla protezione dei dati personali”).

Proseguendo con la compilazione, acconsenti al trattamento dei dati
(se non acconsenti, non potrai partecipare alla ricerca)

- ○ Acconsento
- ○ Non acconsento

Età

____

Genere

- ○ Maschio
- ○ Femmina

Titolo di studio

- ○ Licenza elementare
- ○ Licenza media
- ○ Diploma
- ○ Laurea

Stato occupazionale

- ○ Lavoratore/trice
- ○ Pensionato/a
- ○ Inoccupato/a
- ○ Altro ______________________________

Regione in cui risiedi:

- Abruzzo
- Basilicata
- Calabria
- Campania
- Emilia-Romagna
- Friuli Venezia Giulia
- Lazio
- Liguria
- Lombardia
- Marche
- Molise
- Piemonte
- Puglia
- Sardegna
- Sicilia
- Toscana
- Trentino-Alto Adige
- Umbria
- Valle d'Aosta
- Veneto

Ricevi assistenza neurologica nella stessa Regione in cui risiedi?

- ◯ Sì
- ◯ No

Regione in cui ricevi assistenza neurologica:

- ◯ Abruzzo
- ◯ Basilicata
- ◯ Calabria
- ◯ Campania
- ◯ Emilia-Romagna
- ◯ Friuli Venezia Giulia
- ◯ Lazio
- ◯ Liguria
- ◯ Lombardia
- ◯ Marche
- ◯ Molise
- ◯ Piemonte
- ◯ Puglia
- ◯ Sardegna
- ◯ Sicilia
- ◯ Toscana
- ◯ Trentino-Alto Adige
- ◯ Umbria
- ◯ Valle d'Aosta
- ◯ Veneto

Diagnosi

- ○ Malattia di Parkinson
- ○ Parkinsonismo atipico

Anno in cui hai ricevuto la diagnosi

____

Attualmente, come giudichi la tua condizione motoria?

- ○ Il mio disturbo motorio è minimo o assente ed interessa solo una parte del corpo
- ○ Il mio disturbo motorio interessa entrambe le parti del corpo ma non ho problemi di equilibrio
- ○ Nei cambi di posizione avverto una lieve difficoltà a mantenere l’equilibrio ma questo non interferisce con il mio grado di autonomia
- ○ Cammino con difficoltà e quasi sempre necessito di un aiuto
- ○ Non cammino autonomamente e necessito di aiuto per svolgere tutte le attività quotidiane

---

Hai ricevuto una diagnosi di una o più patologie croniche tra le seguenti?

- ☐ Psichiatriche
- ☐ Decadimento cognitivo
- ☐ No, nessuna delle precedenti

Hai mai avuto la possibilità di eseguire la visita neurologica a distanza prima della pandemia Covid-19 (per es. telefono, SMS, Chat, E-mail, Videoconsulto)?

- ○ Sì
- ○ No

Durante la pandemia Covid-19, hai avuto la possibilità di accedere a soluzioni alternative alla visita neurologica in presenza (per es. telefono, SMS, Chat, E-mail, Videoconsulto)?

- ○ Sì
- ○ No

Hai ricevuto assistenza tecnica da parte di un'altra persona per accedere al consulto neurologico a distanza?

- ○ Sì
- ○ No

Durante la pandemia Covid-19, per quali problematiche hai richiesto un consulto con il neurologo?

- ☐ Visita di controllo periodica
- ☐ Prescrizioni farmacologiche
- ☐ Regolazione dei parametri DBS
- ☐ Comparsa di effetti collaterali ai farmaci (dispercezioni visive, alterazioni del comportamento, ect)
- ☐ Altro ____

Chi ti ha informato della possibilità di eseguire la visita neurologia attraverso un consulto a distanza?

- ○ Medico
- ○ Associazioni di pazienti
- ○ Altri pazienti
- ○ Internet
- ○ Altro ____

Quali strumenti digitali hai utilizzato per accedere al consulto neurologico a distanza? (possibili più risposte)

- ☐ Telefono
- ☐ Email
- ☐ SMS
- ☐ Chat
- ☐ Videoconsulto es. videochiamata in WhatsApp, Skype, Zoom, Microsoft Teams
- ☐ Altro ____

ORA TI CHIEDIAMO DI FOCALIZZARTI SULLA TUA ESPERIENZA DI VIDEOCONSULTO (ES. VIDEOCHIAMATA IN WHATSAPP, SKYPE, ZOOM, MICROSOFT TEAMS) E DI PENSARE AGLI ASPETTI TECNICI. ESPRIMI IL TUO GRADO DI ACCORDO/DISACCORDO CON LE SEGUENTI AFFERMAZIONI

	Per nulla d'accordo	Abbastanza in disaccordo	Né in accordo né in disaccordo	Abbastanza d'accordo	Molto d'accordo
1. Ritengo di avere le competenze informatiche necessarie	◯	◯	◯	◯	◯
2. Ritengo di essere in possesso di strumentazioni informatiche e digitali adeguate	◯	◯	◯	◯	◯
3. Ritengo di aver ricevuto tutte le istruzioni necessarie per l'espletamento del consulto neurologico (password, link, altro)	◯	◯	◯	◯	◯
4. Ritengo che sia stato semplice concordare un appuntamento con il neurologo	◯	◯	◯	◯	◯
5. La qualità dell'audio durante il consulto neurologico è stata adeguata	◯	◯	◯	◯	◯

6. La qualità del video durante il consulto neurologico è stata adeguata	○	○	○	○	○
7. Ogni volta che ho riscontrato problemi tecnici ho risolto facilmente e rapidamente	○	○	○	○	○
8. È stato semplice usare i sistemi digitali per il consulto	○	○	○	○	○
9. È stato facile imparare ad usare i sistemi digitali per il consulto	○	○	○	○	○
10. Credo che potrei diventare sempre più esperto utilizzando servizi digitali per accedere a prestazioni sanitarie	○	○	○	○	○
11. Ritengo che la mia privacy sia stata rispettata	○	○	○	○	○

ORA PENSA ALLA TUA ESPERIENZA DI VIDEOCONSULTO NEUROLOGICO A DISTANZA ED ESPRIMI IL GRADO DI ACCORDO/DISACCORDO CON LE SEGUENTI AFFERMAZIONI

	Per nulla d'accordo	Abbastanza in disaccordo	Né in accordo né in disaccordo	Abbastanza d'accordo	Molto d'accordo
12. Mi ha permesso di risparmiare il tempo evitando di dover raggiungere fisicamente la struttura sanitaria	○	○	○	○	○
13. Mi ha permesso di ridurre i tempi di attesa della visita	○	○	○	○	○
14. Mi ha permesso di risparmiare denaro	○	○	○	○	○
15. Ha migliorato le mie condizioni di salute	○	○	○	○	○
16. Mi ha permesso di essere coinvolto più attivamente nella gestione della mia salute	○	○	○	○	○
17. Sono stato meno preoccupato/a per la mia condizione di salute	○	○	○	○	○
18. Attraverso il consulto a distanza ho compreso adeguatamente le indicazioni farmacologiche prescritte	○	○	○	○	○

19. Attraverso il consulto a distanza ho ricevuto informazioni adeguate sulla mia condizione clinica	○	○	○	○	○
20. Attraverso il consulto a distanza ho ricevuto complessivamente informazioni sufficienti	○	○	○	○	○
21. Quando sarà finita l’emergenza sanitaria, vorrei continuare ad usufruire di tale servizio	○	○	○	○	○
22. Lo consiglierei ad un’altra persona	○	○	○	○	○
23. Mi sono sentito/a a mio agio nel comunicare con il neurologo a distanza	○	○	○	○	○
24. Le informazioni ricevute sono state sufficientemente accurate	○	○	○	○	○

PENSA ALL’INTERAZIONE CHE HAI AVUTO CON IL TUO NEUROLOGO DURANTE IL VIDEOCONSULTO ED ESPRIMI IL GRADO DI ACCORDO/DISACCORDO CON LE SEGUENTI AFFERMAZIONI

	Per nulla d'accordo	Abbastanza in disaccordo	Né in accordo né in disaccordo	Abbastanza d'accordo	Molto d'accordo
25. È stato facile esprimermi in modo efficace durante il consulto	○	○	○	○	○
26. Ho potuto comprendere chiaramente quello che mi diceva il neurologo	○	○	○	○	○
27. Sono riuscito a relazionarmi con il neurologo come se fossimo in una visita in presenza	○	○	○	○	○
28. È stato confortevole ricevere un consulto neurologico senza spostarmi da casa	○	○	○	○	○
29. Mi sono sentito/a a mio agio nell’utilizzare gli strumenti digitali	○	○	○	○	○
30. Mi sono sentito/a a mio agio nell’interagire con il neurologo	○	○	○	○	○

31. Mi ha dato la possibilità di instaurare una buona relazione con il neurologo	○	○	○	○	○
32. Mi sono sentito accolto nonostante la distanza fisica	○	○	○	○	○
33. Il neurologo ha dedicato un tempo adeguato all’ascolto dei miei problemi	○	○	○	○	○
34. Durante il consulto il neurologo ha mostrato un atteggiamento empatico	○	○	○	○	○

PENSA ALLA TUA ESPERIENZA DI CONSULTO NEUROLOGICO A DISTANZA ED ESPRIMI IL TUO GRADO DI SODDISFAZIONE RISPETTO ALLE SEGUENTI DIMENSIONI

	Per nulla soddisfatto	Abbastanza insoddisfatto	Né soddisfatto né insodisfatto	Abbastanza soddisfatto	Molto soddisfatto
35. Qualità complessiva del servizio ricevuto	○	○	○	○	○
36. Attrezzatura digitale utilizzata dal professionista	○	○	○	○	○
37. Attrezzatura digitale da me utilizzata	○	○	○	○	○
38. Puntualità del servizio ricevuto	○	○	○	○	○
39. Utile strumento per ricevere assistenza sanitaria	○	○	○	○	○
40. Attenzione adeguata al mio bisogno di assistenza sanitaria	○	○	○	○	○
41. Tempo intercorso tra la prenotazione ed il consulto	○	○	○	○	○

TI CHIEDIAMO ORA DI PENSARE ALLE DIFFERENZE TRA VISITA NEUROLOGICA IN PRESENZA E CONSULTO NEUROLOGICO A DISTANZA. IN PARTICOLARE, CI INTERESSA CAPIRE PER OGNI ASPETTO PRESO AD ESAME SE SIA MEGLIO UN SERVIZIO IN PRESENZA, IL CONSULTO ONLINE OPPURE SE RITIENI NON CI SIANO DIFFERENZE

	Meglio visita in presenza	Meglio online	Non ci sono differenze
42. Accuratezza della visita	○	○	○
43. Valutazione delle prescrizioni farmacologiche	○	○	○
44. Durata della visita	○	○	○
45. Puntualità della visita	○	○	○
46. Possibilità di essere ascoltati	○	○	○
47. Grado di empatia del professionista	○	○	○
48. Possibilità di fare domande al professionista	○	○	○
49. Facilità di comprensione di quello che dice il professionista	○	○	○
50. Rispetto della privacy	○	○	○
